# A Phase 2 Randomized, Double-Blind, Placebo-Controlled Trial of MHAA4549A, a Monoclonal Antibody, plus Oseltamivir in Patients Hospitalized with Severe Influenza A Virus Infection

**DOI:** 10.1128/AAC.00352-20

**Published:** 2020-06-23

**Authors:** Jeremy J. Lim, Anna C. Nilsson, Michael Silverman, Nimer Assy, Priya Kulkarni, Jacqueline M. McBride, Rong Deng, Chloe Li, Xiaoying Yang, Allen Nguyen, Priscilla Horn, Mauricio Maia, Aide Castro, Melicent C. Peck, Joshua Galanter, Tom Chu, Elizabeth M. Newton, Jorge A. Tavel

**Affiliations:** a Genentech, Inc., South San Francisco, California, USA; b Department of Translational Medicine, Infectious Diseases Research Unit, Lund University, Malmö, Sweden; c London Health Sciences Centre, London, Ontario, Canada; d Galilee Medical Center, Department of Internal Med A, The Azrieli Faculty of Medicine, Nahariya, Israel

**Keywords:** MHAA4549A, antiviral agents, influenza A virus, monoclonal antibody

## Abstract

For patients hospitalized with severe influenza A virus infection, morbidity and mortality remain high. MHAA4549A, a human monoclonal antibody targeting the influenza A virus hemagglutinin stalk, has demonstrated pharmacological activity in animal studies and in a human influenza A challenge study. We evaluated the safety and efficacy of MHAA4549A plus oseltamivir against influenza A virus infection in hospitalized patients. The CRANE trial was a phase 2b randomized, double-blind, placebo-controlled study of single intravenous (i.v.) doses of placebo, 3,600 mg MHAA4549A, or 8,400 mg MHAA4549A each combined with oral oseltamivir (+OTV) in patients hospitalized with severe influenza A virus infection. Patients, enrolled across 68 clinical sites in 18 countries, were randomized 1:1:1. The primary outcome was the median time to normalization of respiratory function, defined as the time to removal of supplemental oxygen support to maintain a stable oxygen saturation (SpO_2_) of ≥95%. Safety, pharmacokinetics, and effects on influenza viral load were also assessed. One hundred sixty-six patients were randomized and analyzed during a preplanned interim analysis. Compared to placebo+OTV, MHAA4549A+OTV did not significantly reduce the time to normalization of respiratory function (placebo+OTV, 4.28 days; 3,600 mg MHAA4549A+OTV, 2.78 days; 8,400 mg MHAA4549A+OTV, 2.65 days), nor did it improve other secondary clinical outcomes. Adverse event frequency was balanced across cohorts. MHAA4549A+OTV did not further reduce viral load versus placebo+OTV. In hospitalized patients with influenza A virus infection, MHAA4549A did not improve clinical outcomes over OTV alone. Variability in patient removal from oxygen supplementation limited the utility of the primary endpoint. Validated endpoints are needed to assess novel treatments for severe influenza A virus infection. (This study has been registered at ClinicalTrials.gov under registration no. NCT02293863.)

## Abstract (precis)

For patients hospitalized with severe influenza A virus infection, morbidity and mortality remain high. MHAA4549A, a human monoclonal antibody targeting the influenza A virus hemagglutinin stalk, has demonstrated pharmacological activity in animal studies and in a human influenza A challenge study. We evaluated the safety and efficacy of MHAA4549A plus oseltamivir against influenza A virus infection in hospitalized patients. The CRANE trial was a phase 2b randomized, double-blind, placebo-controlled study of single intravenous (i.

## INTRODUCTION

Seasonal influenza affects 5% to 10% of the world’s adult population (26), causing three to five million severe cases (1) and approximately 290,000 to 650,000 seasonal influenza-associated respiratory deaths each year (2). Respiratory failure, a hallmark of influenza, is a major driver of hospitalization, morbidity, and mortality. For patients hospitalized with influenza, standard-of-care therapy includes supportive measures and administration of neuraminidase inhibitors (NAIs). NAIs are most effective when administered within 48 h of symptom onset, but patients are often not admitted so early in the course of infection (3–6).

Passive immunization is well established for viral infection prophylaxis, and licensed polyclonal antibody products target cytomegalovirus, hepatitis B virus, and varicella-zoster virus. A recent study of immune plasma plus standard-of-care therapy versus standard-of-care therapy alone to treat severe influenza demonstrated trends toward normalization of respiratory status by day 28 (67% versus 53%; *P* = 0.069) and clinical benefit in secondary outcomes but not statistical significance (7). The limited supply of convalescent plasma and low antibody titers make this approach challenging. It is also nonspecific, given that polyclonal preparations target multiple epitopes. Broadly neutralizing monoclonal antibodies targeting a single epitope may be more viable as treatment for patients with severe influenza (8, 9).

MHAA4549A, a human monoclonal immunoglobulin G1 (IgG1) antibody to influenza A virus, was cloned from a single-human plasmablast cell from an influenza virus-vaccinated donor (5). MHAA4549A binds a highly conserved epitope on the influenza A virus hemagglutinin (HA) stalk, blocking fusion of the viral envelope with the host cell endosomal membrane, thus preventing subsequent viral replication (10). *In vitro*, MHAA4549A neutralizes several human seasonal influenza A virus strains, including H1N1, H3N2, and H2N2 isolates (5). MHAA4549A also binds HA on the surfaces of influenza-infected cells, inducing lysis by natural killer cells via antibody-dependent cell-mediated cytotoxicity (L. Kamen and E. Kho, personal communication).

In *in vivo* animal models, MHAA4549A increased survival over oseltamivir (OTV) and had an additive effect with OTV to significantly reduce mortality over either treatment alone, supporting coadministration of MHAA4549A with OTV (5). In phase 1 healthy-volunteer trials, MHAA4549A was well tolerated as a single intravenous (i.v.) dose up to 10,800 mg (11). In a phase 2a nasal influenza virus healthy-volunteer challenge (HVC) study, a single i.v. dose of 3,600 mg MHAA4549A monotherapy reduced nasopharyngeal viral shedding by an average of 97.5% and peak viral load by an average of 77.3% compared with placebo (12).

Given these findings, we hypothesized that combining MHAA4549A and OTV may benefit patients with severe influenza and potentially prevent viral resistance due to a lack of cross-resistance between OTV and MHAA4549A (10). In this report, we present the results of an interim analysis of the CRANE study (ClinicalTrials registration no. NCT02293863), a phase 2b, randomized, double-blind, placebo-controlled trial to evaluate the safety and efficacy of a single i.v. dose of MHAA4549A plus oral OTV (MHAA4549A+OTV) compared with placebo plus oral OTV (placebo+OTV) in patients hospitalized with severe influenza A virus infection.

## RESULTS

**FIG 1:**
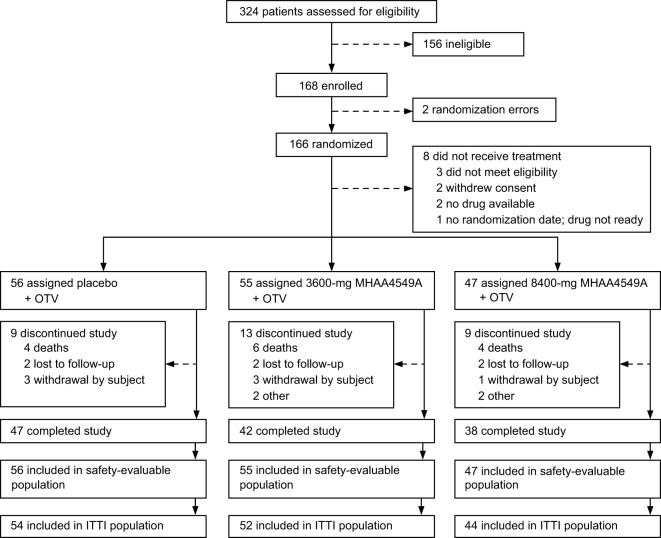
CRANE trial profile. The safety-evaluable population was defined as all subjects who received study treatment. The ITTI population was defined as patients who were positive in a day 1, centrally performed PCR test for influenza A. ITTI, intent-to-treat infected; OTV, oseltamivir.

Screening and randomization of patients occurred from 14 January 2015 through 21 March 2017. We performed this interim analysis after 166 patients were randomized to study treatment, and 158 patients were dosed (Fig. 1). Patients received single i.v. doses of 3,600 mg or 8,400 mg MHAA4549A+OTV or placebo+OTV. The mean age of patients across all groups was 60.7 years (range, 18 to 95 years) (Table 1). The placebo+OTV group was older (median age, 65.7 years) than the 3,600-mg and 8,400-mg MHAA4549A+OTV groups (median ages, 56.5 years and 59.8 years, respectively), with a greater proportion of patients above age 65 and with confirmed or suspected bacterial pneumonia (Table 1). The placebo+OTV group also had a higher proportion of patients with comorbidities and a higher proportion of H3N2 influenza than the MHAA4549A+OTV groups (Table 1).

**TABLE 1:** Patient demographics and baseline characteristics (safety population)^*a*^

Characteristic	Value for group		
	Placebo+OTV (*n* = 56)	3,600 mg MHAA4549A+OTV (*n* = 55)	8,400 mg MHAA4549A+OTV (*n* = 47)
Age (years)	65.7 (17.5)	56.5 (18.2)	59.8 (17.9)
Age group, ≥65 (years)	33 (59%)	14 (26%)	20 (43%)
Sex, female	24 (43%)	25 (46%)	22 (47%)
Race			
American Indian or Alaska	1 (2%)	0	1 (2%)
Asian	4 (7%)	0	2 (4%)
Black or African American	1 (2%)	1 (2%)	0
White	45 (80%)	44 (80%)	39 (83%)
Multiple	0	1 (2%)	0
Unknown	5 (9%)	9 (16%)	5 (11%)
Weight (kg)^*b*^	81 (30.8)	83.7 (27.6)	78.41 (17.4)
Oxygen requirement at randomization			
Oxygen supplement (non-PPV)	38 (68%)	39 (71%)	35 (75%)
PPV	18 (32%)	16 (29%)	12 (25%)
Confirmed or suspected bacterial pneumonia	32 (57%)	24 (44%)	23 (49%)
At least one medical condition	54 (96%)	49 (89%)	43 (92%)
Influenza subtype^*c*^			
H1N1	15/54 (28%)	26/52 (50%)	18/44 (41%)
H3N2	39/54 (72%)	26/52 (50%)	24/44 (55%)
Unknown	0	0	2/44 (4%)

a Ages and weights are given as mean (standard deviation); other values are number (percent) of patients. Abbreviations: *n*, number of subjects; PPV, positive-pressure ventilation.

b Data were not available for all safety-evaluable patients.

c ITTI population; data are number/total (percent) for each cohort.

**FIG 2:**
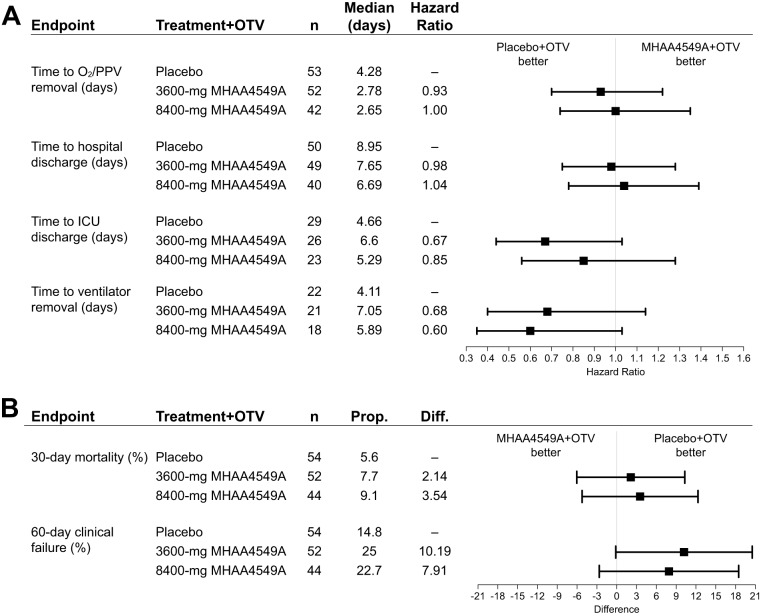
Efficacy endpoints. (A) Hazard ratios (80% CIs) for time-to-event medians were calculated using Wald’s methods, using a stratified analysis based on bacterial pneumonia status (yes or no) and respiratory status (oxygen supplementation or PPV) at randomization. A hazard ratio of >1 favors MHAA4549A treatment. (B) The 80% CIs for the differences (Diff; calculated as treatment − control) between proportions were calculated using Wald’s methods. PPV, positive-pressure ventilation; Prop, proportion.

MHAA4549A+OTV demonstrated a numerical, but not statistically significant, improvement over placebo+OTV in the primary outcome, the median time to normalization of respiratory function (Fig. 2A; also, see Table S1 in the supplemental material): 2.78 days and 2.65 days for the 3,600-mg and 8,400-mg MHAA4549A+OTV groups, respectively, versus 4.28 days for the placebo+OTV group, with hazard ratios (HRs) based on stratified analysis of 0.93 and 1.00, respectively (Table S1). The timing of MHAA4549A administration (within versus after 48 h of symptom onset) had no clear effect on the time to removal of oxygen supplementation (data not shown).

Secondary outcomes showed no statistically significant differences between the MHAA4549A+OTV treatment arms and the placebo+OTV group (Fig. 2; Table S1). The median duration of hospitalization was not significantly different between the three treatment groups (7.65 and 6.69 days versus 8.95 days) (Fig. 2A) and was not reduced further when MHAA4549A was administered within 48 h versus after 48 h of symptom onset. The placebo+OTV group trended toward faster times to ventilator removal and intensive care unit (ICU) discharge, less clinical failure at day 60, and lower 30-day mortality (Fig. 2A and B).

**FIG 3:**
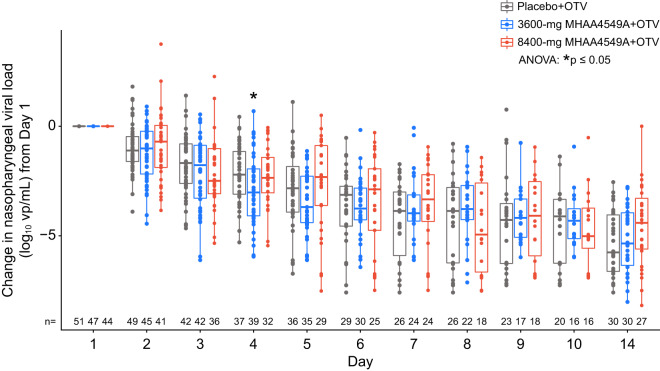
Median change from baseline in nasopharyngeal viral load (measured by quantitative RT-PCR) from day 1 (ITTI population). ANOVA, analysis of variance; OTV, oseltamivir; vp, viral particles. Dots, values for individual subjects; thick bars, medians; boxes, interquartile ranges; whiskers, upper and lower 25% of values.

Using serial nasopharyngeal swab specimens, we analyzed the effect of MHAA4549A on influenza viral burden in the upper respiratory tract with quantitative reverse transcription-PCR (RT-PCR). The overall median change from baseline in viral load was similar across treatment groups (Fig. 3), and the median duration of viral shedding was comparable between the placebo+OTV arm (4 days; range, 0.6 to 26.8), the 3,600-mg MHAA4549A+OTV arm (4.63 days; range, 0.5 to 29.7) and the 8,400-mg MHAA4549A+OTV arm (4.60 days; range, 0.6 to 13.5). Stratified analysis showed no statistical significance in the duration of viral shedding between the active treatment arms and placebo+OTV arm (3,600 mg MHAA4549A+OTV, *P* = 0.9 and HR = 1.00; 8,400 mg MHAA4549A+OTV, *P* = 0.8 and HR = 1.18). Changes in nasopharyngeal viral burden area under the curve (AUC) or peak viral burden did not differ between treatment groups (data not shown).

Serum MHAA4549A concentrations (mean maximum concentration [*C*_max_] range, approximately 900 to 2,000 μg/ml) exhibited a biphasic disposition, with an initial rapid distribution phase followed by a slow elimination phase, typical of i.v. monoclonal IgG. MHAA4549A serum exposure was confirmed in all MHAA4549A-treated patients. The mean terminal half-lives for MHAA4549A were 19.0 ± 4.91 and 17.8 ± 3.88 days, respectively, in the 3,600-mg and 8,400-mg MHAA4549A+OTV groups.

No statistically significant imbalances were observed for safety events between treatment arms (Table 2; Table S2). Overall, 117 patients (74.1%) reported 511 adverse events (AEs) during the study. Of these, 16 patients (10.1%) reported AEs related to MHAA4549A (Table 2). The most common treatment-related AEs were nausea (4 [2.5%] patients) and vomiting (2 [1.3%] patients). Thirty-one patients (19.6%) reported 50 serious AEs (SAEs) (Table 2). SAEs reported by ≥1% of patients included pneumonia (7 [4.4%] patients), septic shock and pulmonary embolism (3 [1.9%] patients each), and acute respiratory distress syndrome, multiple organ dysfunction syndrome, and acute kidney injury (2 [1.3%] patients each). Two SAEs (1.3%), one of hypomagnesemia (placebo+OTV) and one of hypotension (8,400 mg MHAA4549A+OTV), were reported as being related to treatment (Table 2). We observed no clinically significant changes in electrocardiogram (ECG) parameters, laboratory values, or vital signs.

**TABLE 2:** Occurrence of AEs, SAEs, and deaths^*a*^

Patient group (*n*)	Overall total no. of AEs	No. (%) of patients:		Overall total no. of SAEs	No. (%) of patients:		Total no. of deaths
		With ≥1 AE	With study drug-related AEs		With ≥1 SAE	With study drug-related SAEs	
Placebo+OTV (56)	187	45 (80.4%)	5 (8.9%)	13	8 (14.3%)	1 (1.8%)	4 (7.1%)
3,600 mg MHAA4549A+OTV (55)	196	37 (67.3%)	7 (12.7%)	20	11 (20.0%)	0	6 (10.9%)
8,400 mg MHAA4549A+OTV (47)	128	35 (74.5%)	4 (8.5%)	17	12 (25.5%)	1 (2.1%)	4 (8.5%)
All patients (158)	511	117 (74.1%)	16 (10.1%)	50	31 (19.6%)	2 (1.3%)	14 (8.9%)

a Abbreviations: AE, adverse event; SAE, serious adverse event.

Fifty-six patients had grade 3 or 4 AEs. The most common grade ≥3 AEs were in the system organ classes of respiratory, thoracic, and mediastinal disorders (16 patients total) followed by gastrointestinal disorders, infections and infestations, and cardiac disorders (9 patients each), vascular disorders (8 patients), and investigations (7 patients). Two patients reported treatment-related grade 3 AEs: one patient each reported abnormal levels of aspartate aminotransferase (grade 3) and hypomagnesemia (grade 3). One treatment-related SAE of grade 4 hypotension occurred in a 48-year-old female (8,400 mg MHAA4549A+OTV) beginning on day 1, several hours after study drug infusion, and resolved on day 2. Fourteen (8.9%) patients died during the study (Table 2). Thirteen (8.2%) deaths were due to non-treatment-related SAEs; one death was due to the natural course of influenza, according to the site investigator.

Among 158 patients with baseline-evaluable samples, two tested anti-drug antibody (ADA) positive (ADA prevalence = 1.3%). Of 127 postbaseline-evaluable patients, one patient (3,600 mg MHAA4549A+OTV) tested ADA positive both pre- and postbaseline but showed no increase in ADA titer after treatment.

## DISCUSSION

To our knowledge, this is the first published clinical trial of a monoclonal antibody to treat patients hospitalized with severe influenza. Compared with placebo+OTV, MHAA4549A+OTV did not significantly reduce the median time to removal of oxygen supplementation or positive-pressure ventilation (PPV), nor did the combination show statistically significant improvements in secondary efficacy endpoints. Overall, safety was consistent across all treatment groups, including SAEs related to study drug. Nasopharyngeal viral load showed no statistically significant differences between dose groups. Given the lack of impact on efficacy and virological measures in the interim analyses, we determined that MHAA4549A+OTV was unlikely to demonstrate a significant clinical benefit over placebo+OTV and terminated the trial, thus making this interim analysis final.

Previous *in vitro* studies have demonstrated the high specificity and affinity of MHAA4549A for influenza A virus hemagglutinins, its ability to neutralize a wide genetic diversity of influenza A virus isolates, and the lack of naturally occurring, MHAA4549A-resistant influenza A virus variants (5, 10). *In vivo* rodent models and the human challenge study demonstrated MHAA4549A activity, both alone and with OTV (5, 12). MHAA4549A nasopharyngeal exposure was also consistent with observations in the human challenge study (R. Deng, unpublished data). Despite these findings, at the doses tested here, MHAA4549A did not appear to have antiviral activity beyond that of OTV, as demonstrated by the duration of viral shedding, AUC, or peak viral load. However, quantitative RT-PCR may not distinguish viable from neutralized or noninfectious virus, preventing detection of additional reductions. Although we attempted to measure infectious titers in tracheal aspirates, because of very limited sampling and challenges with performing these assays on frozen samples, these data had reduced value for interpreting pharmacological activity (data not shown). Furthermore, in the phase 2a influenza HVC study, results from qRT-PCR and infectious titer assays were highly correlated and demonstrated similar effects in reduction in median viral AUC and peak viral load (12).

Several possibilities may explain why the phase 2a influenza HVC study results did not translate into clinical benefit for hospitalized patients. First, the controlled nasal inoculation used in HVC models is less virulent than naturally occurring strains; also, exposure to variable quantities of potentially more virulent virus may occur at multiple mucosal sites. Second, in contrast to healthy volunteers, patients hospitalized with severe influenza are typically older, with multiple comorbidities and weakened immune responses affecting their ability to clear virus (13). Bacterial coinfection may also confound efficacy. Furthermore, while MHAA4549A reduced viral load in the upper respiratory tract to undetectable levels, virus may still persist in the lower respiratory tract (14, 15). Although data were limited due to sample collection issues, we detected MHAA4549A in tracheal aspirates from the lower respiratory tract (approximately 500 to 600 μg/ml), so lack of exposure is unlikely to account for the lack of efficacy (R. Deng, unpublished data). Perhaps most importantly, while we controlled the interval from infection to treatment at 24 to 36 h in the HVC study, we cannot know the exact length of this interval in natural infections. As an additional exploratory measure, we examined whether MHAA4549A treatment led to changes in the levels of selected proinflammatory cytokines in serum, but we observed no differences between the MHAA4549A+OTV and placebo+OTV arms (data not shown). Therefore, the totality of the data suggests that treatment may have come too late to reduce viral load or inflammation further than OTV alone. These factors may create a considerably higher treatment bar for overcoming more established viral infections in hospitalized patients.

As in similar influenza trials conducted in a hospitalized population, we faced challenges regarding patient recruitment despite enrollment over 5 influenza seasons (3 in the Northern Hemisphere and 2 in the Southern Hemisphere), with the seasonality of influenza and regional differences for hospital admission criteria contributing to slow recruitment (6–8, 16). Consistent with other hospital-based trials (17), only 40% (68/172) of the clinical sites enrolled one or more subjects. The study design also likely contributed to recruitment challenges by limiting MHAA4549A dosing to within 5 days of symptom onset, within 3 days of initial OTV treatment, and no later than 48 h after hospital admission. This treatment window was based on data demonstrating that hospitalized influenza patients benefit from NAI treatment as late as 5 days from symptom onset (4) and on data from the phase 3 zanamivir trial (18). Additionally, *in vivo* studies had demonstrated MHAA449A efficacy at 72 h postinfection (5); we therefore aimed to assess a prolonged window for initiation of MHAA4549A treatment beyond the current standard of care (48 h from symptom onset). While we believed that the treatment window was a feasible way to enroll patients, recruitment remained challenging. Furthermore, as mentioned above, MHAA4549A may not have been given early enough relative to the onset of infection; however, subgroup analyses based on time to treatment showed no clear trends (data not shown).

There are currently no validated clinical endpoints for assessing anti-influenza virus agents in hospitalized patients (6, 18–21). Virologic endpoints are not considered appropriate because no demonstrated relationship exists between the timing and magnitude of viral reductions and measurable, clinically meaningful improvement (19). Although all-cause mortality is an unambiguous primary endpoint and is required by the U.S. Food and Drug Administration (FDA) for clinical trials in patients with hospital- or ventilator-acquired pneumonia, it has significant limitations, and distinguishing attributable from nonattributable mortality is difficult. For example, underlying comorbidities and supportive care confound mortality rates. Also, demonstrating mortality requires unfeasibly large study sample sizes to yield statistically and clinically meaningful data (21).

The primary endpoint was the time to normalization of respiratory function, defined as oxygen saturation of ≥95% without oxygen supplementation. We hypothesized that time on oxygen or PPV was a clinically meaningful outcome in hospitalized patients that measures a serious consequence of influenza, reflects the severe impact on patient function, and is consistent with clinically relevant endpoints in the FDA guidance for influenza drug development (19). However, collecting primary endpoint data was challenging because the study design lacked an oxygen weaning protocol, and the standard of care for the appropriate time to remove oxygen, including delayed or early discontinuation of supplemental oxygen, varied across the 68 clinical sites in 18 countries due to regional practice differences (21). For clinical feasibility, pulse oximetry was used to determine a patient’s oxygen requirement, but multiple factors can affect this method (22–24). More invasive techniques, such as measuring arterial blood gases, are more accurate, but their frequent implementation imposes too high a patient burden.

In conclusion, the CRANE study demonstrated that MHAA4549A+OTV treatment in patients hospitalized with severe influenza A virus infection does not improve clinical outcomes relative to placebo+OTV. Consensus on a valid primary endpoint may facilitate future trial execution in this patient population with a high, unmet medical need.

## MATERIALS AND METHODS

### Study design.

The CRANE study was a phase 2b randomized, double-blind, placebo-controlled clinical trial to assess the safety and clinical activity of single i.v. doses of 3,600 mg or 8,400 mg MHAA4549A (Genentech, Inc.) plus oral OTV compared with i.v. placebo plus oral OTV in hospitalized patients with severe influenza A virus infection. MHAA4549A, diluted in 0.9% normal saline, was administered by i.v. infusion over ∼120 min. Patients began study drug infusion within 48 h of hospital admission and within 5 days of onset of influenza symptoms (including fever, chills, malaise, dry cough, loss of appetite, myalgias, coryza, or nausea). OTV (75 mg or 150 mg twice daily) was administered orally for a minimum of 5 days starting on day 1, beginning ≤12 h after completion of study drug administration. Follow-up was 60 days from MHAA4549A administration. Randomization, blinding, and dose selection details are provided in the supplemental material.

The CRANE trial was conducted in full conformance with the International Council for Harmonisation (ICH) E6 guideline for good clinical practice (GCP) and the principles of the Declaration of Helsinki, or the laws and regulations of the country where the research was conducted, whichever afforded the greater protection to the individual. The study complied with ICH E2A guidelines (Clinical Safety Data Management: Definitions and Standards for Expedited Reporting). Studies conducted in the European Union (EU) or European Economic Area complied with the EU Clinical Trial Directive (2001/20/EC). All patients or their legally authorized representatives provided written, informed consent.

### Patients.

Study participants were enrolled across 68 investigational clinical sites in 18 countries. Patients aged ≥18 years were eligible if they were positive for influenza A virus, as determined by a local influenza virus antigen test or PCR assay, and required supplemental oxygen to maintain oxygen saturation (pulse oximetry [SpO_2_], >92%) or required PPV within 24 h of hospital admission. Exclusion criteria included three or more days of anti-influenza virus therapy, hospital admission more than 48 h before study treatment, influenza symptom onset more than 5 days before study treatment, and significant immune suppression.

### Outcomes.

The primary endpoint was the median time to normalization of respiratory function, defined as the time to removal of supplemental oxygen support and maintenance of a stable SpO_2_ of ≥95%. Key secondary efficacy outcomes included time to hospital discharge, time to ICU discharge, all-cause mortality at day 30, time to ventilator removal, and clinical failure at day 60, defined as requiring increased oxygen (an increase in oxygen supplementation from low-flow oxygen [2 to 6 liters/min] to high-flow oxygen [>6 liters/min], or from oxygen supplementation alone to PPV or extracorporeal membrane oxygenation) anytime during hospitalization, progression to the ICU, oxygen support over 2 weeks in duration, or death.

Virology outcomes included influenza viral load in nasopharyngeal samples, measured using a validated, quantitative reverse transcription-PCR (RT-PCR) assay (Viroclinics, the Netherlands) to determine the extent (peak viral load and AUC of viral load) and the median duration of viral shedding (12). Pharmacokinetic (PK) outcomes included MHAA4549A serum concentration and mean terminal half-life, as data allowed, in MHAA4549A-treated patients. Safety outcomes included serious and nonserious AEs, laboratory values, vital signs, 12-lead ECGs, and ADAs during and after treatment administration. Details on endpoint and sample collection procedures are provided in the supplemental material.

### Statistical analysis. (i) Sample size determination.

This study aimed to estimate the effect size and generate a hypothesis regarding the effect of MHAA4549A+OTV versus OTV alone. For sample size calculations, the median time to normalization of respiratory function in the control arm was assumed to be 5 days based on the U.S. Premier Database (2007–2012) (see https://products.premierinc.com/downloads/PremierHealthcareDatabaseWhitepaper.pdf). A sample size of 330 patients (110 patients per arm) with a two-sided significance level (α) of 0.2 had 71% power to detect a treatment difference of 1 day or greater in the time to normalization of oxygen status, assuming no difference in efficacy between the two active treatment groups.

### (ii) Interim safety and efficacy analyses.

A protocol-permitted interim analysis of safety and efficacy data was performed after 166 patients were randomized to treatment and had evaluable safety data. Safety analyses included data from all randomized patients who received study drug. Primary and secondary efficacy analyses were performed on the intent-to-treat infected (ITTI) population, which included all randomized patients with influenza, confirmed by PCR at a central laboratory. Patients were grouped according to treatments assigned at randomization. Time-to-event data were analyzed using the Kaplan-Meier method and stratified Cox proportional hazards models and summarized using *n*, medians when estimable, HRs, and 80% confidence intervals (CIs). Patients lost to follow-up were censored at the time they were last known to be on oxygen supplementation or PPV. The difference of proportions and corresponding 80% CIs between treatment groups was estimated using the stratum-adjusted Mantel-Haenszel method (25). For continuous endpoints, analysis-of-covariance methods (after appropriate transformation of data) were used to estimate treatment differences and 80% CIs. A stratified analysis was performed using the following variables: bacterial pneumonia status (confirmed/suspected or none) and oxygen requirement at randomization (oxygen supplementation or PPV at randomization). Details for statistical analyses of virology, serum MHAA4549A PK parameters, and immunogenicity are provided in the supplemental material.

All statistical analyses were performed using SAS (version 9.2) and R (version 3.3.2).

### Data availability.

Qualified researchers may request access to individual patient-level data through the Vivli Center for Global Clinical Research Data (https://vivli.org/). Further details on Roche’s criteria for eligible studies are available (https://clinicalstudydatarequest.com/Study-Sponsors/Study-Sponsors-Roche.aspx). For further details on Roche’s Global Policy on the Sharing of Clinical Information and how to request access to related clinical study documents, see https://www.roche.com/research_and_development/who_we_are_how_we_work/clinical_trials/our_commitment_to_data_sharing.htm.

## Supplementary Material

Supplemental file 1
