# Supplementary material for: A Phase 2 Randomized, Double-Blind, Placebo-Controlled Trial of MHAA4549A, a Monoclonal Antibody, plus Oseltamivir in Patients Hospitalized with Severe Influenza A Virus Infection
Source: Antimicrob Agents Chemother. 2020 Jun 23;64(7):e00352-20. doi: 10.1128/AAC.00352-20 (PMC7318030; doi:10.1128/AAC.00352-20)
Supplement: Supplemental file 1 [file AAC.00352-20-s0001.pdf]

SUPPLEMENTAL MATERIAL

METHODS

Randomization and blinding

Randomization was managed by a central Interactive Voice and Web Response System (IxRS). Patients were randomly assigned to receive 3600-mg MHAA4549A+OTV, 8400-mg MHAA4549A+OTV, or placebo+OTV at a 1:1:1 ratio, and were stratified by country, source of supplemental oxygen (PPV versus nasal canula), and presence of bacterial pneumonia (assessed as either suspected by the site principal investigator or confirmed by microbiological culture or chest X-ray).

An internal monitoring committee (IMC), consisting of two external experts and sponsor representatives not involved in the conduct of the study nor having any contact with study investigators or site staff, reviewed unblinded safety data approximately every 16 weeks during the study to assess whether study data suggested a significant drug-related toxicity or worsening of the disease associated with the study drug. All other sponsor study team members remained blinded throughout the trial. Unblinded site personnel prepared IV infusions of study drug and blinded study personnel administered the study drug. Patients and blinded site personnel remained blinded to patient-specific treatment assignments until final database lock. Pharmacokinetic (PK) samples were collected from patients assigned to the placebo arm to maintain the blinding of treatment assignment, but were not analyzed. Personnel performing PK sample assays were unblinded.

Dose Selection

The 3600-mg MHAA4549A dose was selected based on the Phase 2a influenza challenge study (S1, S2) that demonstrated both a significant decrease in viral shedding in the upper respiratory tract and a decrease in the area-under-the-curve (AUC) of symptom scores in patients who received 3600-mg MHAA4549A compared with patients who received placebo. The CRANE study also investigated an 8400-mg dose based on the hypothesis that severely ill patients hospitalized with influenza infection are likely to have high viral loads and longer durations of viral shedding and might benefit from increased doses of MHAA4549A. Simulations from a semi-quantitative PK model developed from the Phase 2a challenge study suggested that 8400 mg was the minimum dose that would show a separation of nasal exposure from a dose of 3600 mg (S1, S3).

Endpoint and Sample Collection

Oxygen saturation (SpO_2) readings were recorded daily through pulse oximetry. Unless clinically contraindicated, patients on low-flow oxygen (i.e., 2–6 L/min) had a daily trial off supplemental oxygen between 6:00 am and 12:00 pm; SpO_2 was recorded both on and off oxygen supplementation. If the patient was removed from supplemental oxygen outside this time window, the time and date of oxygen removal were recorded.

Serum samples for MHAA4549A PK measurements were collected on Day 1 (30 minutes before MHAA4549A dosing and 60 minutes after the end of infusion), and Days 2, 3, 5, 7, 14, 30, 60 (at study completion or early discontinuation), and at hospital discharge. MHAA4549A serum concentrations were measured using a validated quantitative immunoassay (S4).

Viral load in the upper respiratory tract was measured in nasopharyngeal samples collected from both nostrils (12). Day 1 nasopharyngeal samples were collected immediately prior to study drug infusion. Samples were also collected on Days 2–10, 14, 20, 25, 30, 60 (at study completion or early discontinuation), and at hospital discharge. Viral load was measured using a validated quantitative reverse transcription PCR (RT-PCR) assay with an LLOQ of 2.05 log_{10} viral particles/mL (Viroclinics, Netherlands). Threshold cycle (Ct) values were derived from electron microscopy viral particle counts of several influenza strains and were not normalized by cellular content.

Serum samples for anti-drug antibody (ADA) testing were obtained at baseline, before dosing, and at Days 30 and 60 after MHAA4549A dosing. The presence of ADAs was detected with a validated MHAA4549A-specific bridging ELISA using a two-tier testing approach of screening and confirmatory assays, as previously described (4).

Statistical Analyses: Nasopharyngeal Viral Load, PK, and Immunogenicity
Nasopharyngeal influenza viral load was measured by qPCR in individual patients at baseline and at all available post-treatment timepoints. While sample collection was planned to be daily for multiple timepoints post treatment, not all daily samples were collected. Changes in the extent and duration of viral infection were analyzed relative to each baseline measure for a given patient. These outcomes included peak viral load, AUC of viral load, and duration of viral shedding.

Estimates for serum PK parameters were summarized and interpatient variability was evaluated. Serum concentrations for MHAA4549A at specified PK sampling timepoints were summarized by cohort and visit, respectively.

Immunogenicity analyses included patients with at least one predose and one postdose ADA assessment, with patients grouped according to treatment received. The assessment included patients with at least one ADA sample available for testing. Numbers and proportions of ADA-positive patients and ADA-negative patients during the treatment and follow-up periods were summarized. Patients were considered to be ADA-positive if they i) were ADA-negative at baseline and tested ADA-positive at any subsequent timepoint (treatment-induced ADA response), or ii) if they were ADA-positive at baseline and had a ≥4-fold increase in ADA titer at any subsequent timepoint (treatment-enhanced ADA response).

REFERENCES

S1. McBride JM, Lim JJ, Burgess T, et al. Phase 2 randomized trial of the safety and efficacy of MHAA4549A, a broadly neutralizing monoclonal antibody, in a human influenza A virus challenge model. Antimicrob Agents Chemother, **2017**; 61: pii: e01154-17. doi: 10.1128/AAC.01154-17.

S2. Deng R, Lee AP, Maia M, et al. Pharmacokinetics of MHAA4549A, an anti-influenza A monoclonal antibody, in healthy subjects challenged with influenza a virus in a phase IIa randomized trial. Clin Pharmacokinet **2018**; 57: 367–377. doi: 10.1007/s40262-017-0564-y.

S3. Patel K, Kirkpatrick C, Stroh M, Smith P, Deng R. Population pharmacokinetic/pharmacodynamic (PK/PD) modeling of MHAA4549A, an anti-influenza A monoclonal antibody in healthy subjects challenged with influenza A virus [poster no. A-053]. In: Presented at the 55th Interscience Conference on Antimicrobial Agents and Chemotherapy (ICAAC) Annual Meeting. San Diego; **2015**. Available at http://www.abstractsonline.com/Plan/ViewAbstract.aspx?sKey=066fe91c-1dff-48d2-a97e-9d682ce3a71c&cKey=e5865157-b5ac-4d2a-b813-d30a149af1e5&mKey=7a574a80-eab1-4b50-b343-4695df14907e. Accessed 1 Oct 2016 (17–21 Sep 2015).

S4. Lim JJ, Deng R, Derby MA, et al. Two phase 1, randomized, double-blind, placebo-controlled, single-ascending-dose studies to investigate the safety, tolerability, and pharmacokinetics of an anti-influenza A virus monoclonal antibody, MHAA4549A, in healthy volunteers. Antimicrob Agents Chemother, **2016**; 60: 5437-5444. doi: 10.1128/AAC.00607-16.

RESULTS

TABLE S1 Primary and Secondary Efficacy Endpoints

Endpoint	Parameter	Placebo+OTV	3600-mg MHAA4549A+OTV	8400-mg MHAA4549A+OTV
Time to O_2/PPV removal (days)[a]	Median, 80% CI[b]	4.28 (3.06, 6.60)	2.78 (2.52, 4.20)	2.65 (1.58, 4.52)
	HR (80% CI)[c]		0.93 (0.70, 1.22)	1.00 (0.74, 1.35)
	n, p-value[d]	53	52, 0.40	42, 0.33
Time to hospital discharge (days)[a]	Median (80% CI)[b]	8.95 (5.9, 10.29)	7.65 (6.94, 8.02)	6.69 (6, 8.86)
	HR (80% CI)[c]		0.98 (0.75, 1.28)	1.04 (0.78, 1.39)
	n, p-value[d]	50	49, 0.61	40, 0.99
Time to ICU discharge (days)[a]	Median (80% CI)[b]	4.66 (3.91, 7.1)	6.6 (4.82, 10.53)	5.29 (3.25, 6.58)
	HR (80% CI)[c]		0.67 (0.44, 1.03)	0.85 (0.56, 1.28)
	n, p-value[d]	29	26, 0.13	23, 0.73
Time to ventilation removal (days)[a]	Median (80% CI)[b]	4.11 (2.72, 5.32)	7.05 (1.92, 13.12)	5.89 (4.13, 13.07)
	HR (80% CI)[c]		0.68 (0.40, 1.14)	0.6 (0.35, 1.03)
	n, p-value[d]	22	21, 0.30	18, 0.52
30-day mortality (%)	% (80% CI)[e]	5.6 (2.06, 11.95)	7.7 (3.40, 14.79)	9.1 (4.02, 17.35)
	Diff (80% CI)[f]		2.14 (-6.04, 10.31)	3.54 (-5.24, 12.31)
	n, p-value[g]	54	52, 0.66	44, 0.50
60-day clinical failure (%)	% (80% CI)[e]	14.8 (8.82, 22.95)	25.0 (17.22, 34.32)	22.7 (14.63, 32.84)
	Diff (80% CI)[f]		10.19 (-0.15, 20.52)	7.91 (-2.64, 18.47)
	n, p-value[g]	54	52, 0.19	44, 0.32

Abbreviations: CI, confidence interval; Diff, difference in event rates (Diff = treatment – control); HR, hazard ratio; ICU, intensive care unit; OTV, oseltamivir; PPV, positive pressure ventilation.

[a]HRs, 80% CIs, and p-values were calculated using a stratified analysis based on bacterial pneumonia status (yes or no) and respiratory status (oxygen supplementation or PPV) at randomization.

Calculated using [b]Brookmeyer-Crowley, [c]Wald's, and [d]Wilcoxon methods respectively. Hazard ratio (HR) was defined as treatment/control where >1 is in favor of treatment.

Calculated using [e]Pearson-Clopper, [f]Wald's, and [g]Cochran-Mantel-Haenszel methods, respectively.

TABLE S2 AEs in >2% of All Patients

Preferred term	Placebo+OTV (n=56)	3600-mg MHAA4549A+OTV (n=55)	8400-mg MHAA4549A+OTV (n=47)	All patients (n=158)
Hypertension	7 (12.5%)	1 (1.8%)	4 (8.5%)	12 (7.6%)
Pneumonia	2 (3.6%)	4 (7.3%)	5 (10.6%)	11 (7.0%)
Hypokalemia	4 (7.1%)	2 (3.6%)	4 (8.5%)	10 (6.3%)
Diarrhea	7 (12.5%)	3 (5.5%)	0	10 (6.3%)
Atrial fibrillation	4 (7.1%)	4 (7.3%)	2 (4.3%)	10 (6.3%)
Nausea	4 (7.1%)	4 (7.3%)	1 (2.1%)	9 (5.7%)
Hypophosphatemia	4 (7.1%)	2 (3.6%)	2 (4.3%)	8 (5.1%)
Constipation	2 (3.6%)	4 (7.3%)	2 (4.3%)	8 (5.1%)
Pyrexia	2 (3.6%)	4 (7.3%)	2 (4.3%)	8 (5.1%)
Agitation	4 (7.1%)	3 (5.5%)	1 (2.1%)	8 (5.1%)
Hematuria	3 (5.4%)	5 (9.1%)	0	8 (5.1%)
Anemia	3 (5.4%)	3 (5.5%)	2 (4.3%)	8 (5.1%)
Vomiting	3 (5.4%)	2 (3.6%)	2 (4.3%)	7 (4.4%)
Headache	2 (3.6%)	2 (3.6%)	3 (6.4%)	7 (4.4%)
Hyperglycemia	1 (1.8%)	3 (5.5%)	2 (4.3%)	6 (3.8%)
Hyperkalemia	1 (1.8%)	1 (1.8%)	3 (6.4%)	5 (3.2%)
Hypoglycemia	1 (1.8%)	3 (5.5%)	1 (2.1%)	5 (3.2%)
Hypomagnesemia	3 (5.4%)	1 (1.8%)	1 (2.1%)	5 (3.2%)
Abdominal pain	2 (3.6%)	1 (1.8%)	2 (4.3%)	5 (3.2%)
Dyspnea	1 (1.8%)	1 (1.8%)	3 (6.4%)	5 (3.2%)
Hypotension	2 (3.6%)	1 (1.8%)	2 (4.3%)	5 (3.2%)
Back pain	1 (1.8%)	2 (3.6%)	2 (4.3%)	5 (3.2%)
Pain in extremity	2 (3.6%)	2 (3.6%)	1 (2.1%)	5 (3.2%)
Hypernatremia	1 (1.8%)	2 (3.6%)	1 (2.1%)	4 (2.5%)
Viral upper respiratory tract infection	2 (3.6%)	1 (1.8%)	1 (2.1%)	4 (2.5%)
Acute respiratory distress syndrome	1 (1.8%)	2 (3.6%)	1 (2.1%)	4 (2.5%)
Pulmonary embolism	0	2 (3.6%)	2 (4.3%)	4 (2.5%)
Tachycardia	2 (3.6%)	1 (1.8%)	1 (2.1%)	4 (2.5%)
Acute kidney injury	2 (3.6%)	1 (1.8%)	1 (2.1%)	4 (2.5%)
Thrombocytosis	1 (1.8%)	1 (1.8%)	2 (4.3%)	4 (2.5%)
Rash	2 (3.6%)	1 (1.8%)	1 (2.1%)	4 (2.5%)

Data are n (%) unless otherwise indicated.

Abbreviations: AE, adverse event; OTV, oseltamivir.
